# Ubp43 gene expression is required for normal Isg15 expression and fetal development

**DOI:** 10.1186/1477-7827-5-13

**Published:** 2007-03-26

**Authors:** Lea A Rempel, Kathleen J Austin, Kenneth J Ritchie, Ming Yan, Meifeng Shen, Dong-Er Zhang, Luiz E Henkes, Thomas R Hansen

**Affiliations:** 1 Department of Animal Science, University of Wyoming, Laramie, Wyoming, 82071, USA; 2 Currently Institute of Maternal-Fetal Biology and the Division of Cancer & Developmental Biology, Department of Pathology and Laboratory Medicine, University of Kansas Medical Center, Kansas City, Kansas 66160, USA; 3 Department of Molecular and Experimental Medicine, The Scripps Research Institute, La Jolla, California 92037, USA; 4 Department of Biomedical Sciences, Animal Reproduction and Biotechnology Laboratory, Colorado State University, Fort Collins, Colorado 80523, USA

## Abstract

**Background:**

Isg15 covalently modifies murine endometrial proteins in response to early pregnancy. Isg15 can also be severed from targeted proteins by a specific protease called Ubp43 (Usp18). Mice lacking Ubp43 (null) form increased conjugated Isg15 in response to interferon. The Isg15 system has not been examined in chorioallantoic placenta (CP) or mesometrial (MM) components of implantation sites beyond 9.5 days post coitum (dpc). It was hypothesized that deletion of Ubp43 would cause disregulation of Isg15 in implantation sites, and that this would affect pregnancy rates.

**Methods:**

Heterozygous (het) Ubp43 mice were mated and MM and CP implantation sites were collected on 12.5 and 17.5 days post-coitum (dpc).

**Results:**

Free and conjugated Isg15 were greater on 12.5 versus 17.5 dpc in MM. Free and conjugated Isg15 were also present in CP, but did not differ due to genotype on 12.5 dpc. However, null CP had greater free and conjugated Isg15 when compared to het/wt on 17.5 dpc. Null progeny died in utero with fetal genotype ratios (wt:het:null) of 2:5:1 on 12.5 and 2:2:1 on 17.5 dpc. Implantation sites were disrupted within the junctional zone and spongiotrophoblast, contained less vasculature based on lectin B4 staining and contained greater Isg15 mRNA and VEGF protein in Ubp43 null when compared to wt placenta.

**Conclusion:**

It is concluded that Isg15 and its conjugates are present in implantation sites during mid to late gestation and that deletion of Ubp43 causes an increase in free and conjugated Isg15 at the feto-maternal interface. Also, under mixed genetic background, deletion of Ubp43 results in fetal death.

## Background

Interferon stimulated gene product 15 (Isg15) is a ubiquitin-like protein that is transiently produced in the uterus during early pregnancy in several species including primates [1], ruminants [2-4], pigs [5] and mice [6,7]. Isg15 post-translationally modifies other proteins through covalent attachment using a mechanism that is similar to, yet distinct from ubiquitinylation. This conjugation (Isgylation) to target proteins involves three classes of enzymes, an E1 and several E2s and E3s. Isg15 functions as a ubiquitin homolog to regulate general cellular processes such as RNA splicing, chromatin remodeling/polymerase II transcription, cytoskeleton organization and regulation, stress responses, and translation by conjugating to and regulating intracellular proteins [8-14]. It also exists in a non-conjugated form and has been shown to be released from cells and to have a cytokine-like role [15,16].

Within ruminant species, conceptus (embryo proper and surrounding membranes)-secreted interferon (IFN)-tau (τ) interacts with type I IFN receptors on the endometrial surface to activate the Janus kinase-signal transducers and activators of transcription (JAK-STAT) signal cascade [17,18]. Activation of the JAK-STAT pathway leads to the increased production of Isg15. In mice and humans, a conceptus-secreted IFN has not been identified, however Isg15 is up-regulated in the uterus in these species as well [1,6,7,19,20]. For example, Isg15 is up-regulated 3.9-fold in human decidualized stromal cells by embryo-secretory products [20]. Furthermore, induction of Isg15 in mouse decidua might be due to an interferon-like cytokine that is released by trophoblast giant cells [7].

Cleavage of ubiquitin from target proteins plays an important role in numerous biological events such as; proteasome-mediated protein degradation [21], preimplantation embryo development [22], cell growth and differentiation [23-25], transcriptional activation [23], and signal transduction [26]. There are at least five distinct families of deubiquitinating enzymes with four subfamilies that are commonly referred to as cysteine proteases [27]. Ubiquitin processing proteases (Ubps/Usps) are the largest and most diverse group within the cysteine protease subfamilies. Sequence homology is limiting among Ubps, however short consensus sequences surround conserved cysteine and histidine residue boxes [26-28]. Diversity in these amino acids may provide specificity for ubiquitin and ubiquitin-like proteases.

Ubp43 (Usp18) is an Isg15-specific protease [29,30] that is up-regulated in response to IFN or lipopolysaccharide [31,32]. Overexpression of Ubp43 in monocyte cells inhibits cytokine-induced terminal differentiation [29]. Chemistry-based proteomics were used to identify potential ubiquitin and ubiquitin-like proteases, which revealed that Ubp43 was specific to Isg15 [33,34].

Mice that survive to birth with a disrupted Ubp43 gene had increased Isg15 conjugation and a decreased life expectancy due to hydrocephalus and associated neurodegenerative disease [30]. These data support the requirement of proper regulation of Isgylation by Ubp43 cleavage. However, more recent experiments suggest that Ubp43 regulates IFN signaling independent of its isopeptidase activity towards Isg15 [35]. The data indicate that Ubp43 binds directly to IFN receptor (AR2 subunit) and inhibits receptor-associated JAK activity. Also, Isg15 -/- and Isg15 E1 (Ube1L) -/- mice that survived to term were reported to be viable and fertile and had no obvious abnormalities [36,37]. Furthermore, the major phenotypes of Ubp43 deficient mice were not rescued in Isg15/Ubp43 or Ube1L/Ubp43 double knockout mice [37,38].

**Figure 1:**
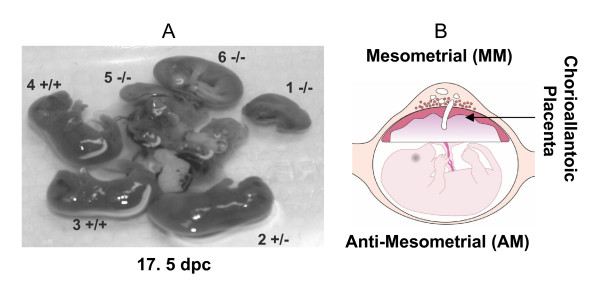
Illustration of fetal development and Ubp43 genotype on day 17.5 of pregnancy (A) and description of how implantation sites were collected (B). A litter from a day 17.5 pregnant mouse shows that all Ubp43 null offspring were dead. Genotype ratio was 2:2:1 (wt:het:null) on day 17.5. On day 12.5, 75% of Ubp43 null mice were dead with a genotype ratio of 2:5:1. There also was a significant loss of +/- fetuses between 12.5 and 17.5 suggesting that Ubp43 gene dosage may also be involved. Panel B shows a cross-sectional representation of a mouse uterus illustrating the various tissues collected for protein and RNA analysis. Anti-mesometrial (AM) represents uterine tissue surrounding the fetal compartment. Fetal chorioallantoic placenta (CP) represents fetal-derived placental tissue. Mesometrial decidua (MM) represents uterine tissue in direct contact with fetal-derived placenta.

Isg15 is localized to antimesometrial decidua during implantation in mice [6,7]. Implantation, which involves adhesion of trophoblast with the endometrium that is followed by invasion, vascularization and placentation begins 4.5–7.5 days post-coitum (dpc) on the antimesometrial pole when the decidualized stroma essential serves as a maternal placenta. The primary adhesion, or attachment occurs on the avascular antimesometrial pole. Later, decidualization extends to the vascular mesometrial pole to facilitate development of the placenta (Figure 1). We first localized Isg15 to the antimesometrial pole of the implantation site [6]. Currently, it is unknown if Isg15 is expressed later than 9.5 dpc in the antimesometrial pole, or if it also appears in the mesometrial decidua and placenta.

A description of embryo development and implantation sites in Ubp43 -/- mice is lacking. Because Isg15 and its conjugates are transiently produced in the uterus during pregnancy in several mammalian species it was theorized that placental development and function might be impaired by Ubp43 deletion. Therefore, the objective was to determine if altered fetal Ubp43 gene expression in a heterozygous uterine environment affected the Isg15 system of maternal and fetal tissues and whether this resulted in lowered fetal viability.

## Methods

### Breeding and tissue collection

Experimental procedures using mice were reviewed and approved by the University of Wyoming Institutional Animal Care and Use Committee. Heterozygous (Ubp43+/-) C57 BL/6 × 129 × Swiss Webster males and females [30] were mated to produce wild-type (wt), heterozygous (+/-) and null (-/-) fetuses within a heterozygous uterine environment. Presence of a vaginal plug on the morning after pairings was designated as 0.5 dpc. Tissues were collected on 12.5 (4 litters; 30 fetuses) or 17.5 (6 litters; 35 fetuses) dpc. Maternal and fetal tissues consisted of uterine tissue at the placental interface or mesometrial (MM) tissue, uterine tissue surrounding the fetus or anti-mesometrial (AM) tissue, and the fetal-derived placental tissue referred to as fetal chorioallantoic placenta (CP) (Figure 1).

At the time of tissue collection a portion of fetal tissue was also collected to determine genotype by PCR techniques. Briefly, tissue was lysed per DNeasy (Qiagen Inc., Valencia, CA) protocol to acquire genomic DNA. Genotyping by PCR technique was conducted using the following primers; Ubp43 exon 2 anti-sense strand, 5'-GCCTGGAAGTGAAGTTGTGGACTCCCG-3'; Ubp43 exon 2 sense strand, 5'-CCAGCGTGAGTACTGCTGCGGCTCAG-3'; and β-galactosidase sense strand, 5'-CGTAACCGTGCATCTGCCAGTTTGAGG-3'. Amplification of a 150 bp fragment resulted from a wt allele and a 370 bp fragment represented a null allele (data not shown). PCR conditions were; 95°C – 2 minutes; (95°C – 15 seconds, 60°C – 15 seconds and 72°C – 30 seconds) × 30 cycles; 72°C – 2 minute extension.

### Protein analysis

Tissue lysates from maternal (MM and AM tissue 12.5 dpc: n = 4 wt, 2 het, and 2 null; 17.5 dpc: n = 4 wt, 4 het, and 2 null) and fetal compartments (CP tissue 12.5 dpc: n = 4 wt, 2 het, and 2 null; 17.5 dpc: n = 4 wt, 4 het, and 2 null) were prepared by homogenizing tissue (100 mg/ml) in 1× Laemmli buffer [39]. Equal concentrations of lysates (12.5 μg per lane) were loaded onto 1D-SDS PAGE gels, electrophoretically transferred to 0.2 μ nitrocellulose and western blot detection was performed using rabbit polyclonal antibodies [anti-mouse Isg15 antibody (1:30,000; [6], anti-human VEGF antibody (1:500; sc-152, Santa Cruz Biotechnology, Inc., Santa Cruz, CA), anti-mouse Angiopoietin-1 antibody (1:1,000; ab8451, Abcam Inc., Cambridge, MA), anti-mouse VEGF Flt-1 Receptor antibody (1:500; sc-316, Santa Cruz Biotechnology, Inc., Santa Cruz, CA), and anti-mouse AMP kinase antibody (1:1,000; A1475-01B, US Biological, Swampscott, MA)]. Secondary antibodies against rabbit were conjugated to either alkaline phosphatase or horseradish peroxidase (Promega, Madison, WI) for visualization of bands. A no-first antibody was used as a control to identify non-specific bands. Detection of a secondary antibody reactive protein was used to verify loading. No differences were seen among samples (P > 0.10; data not shown). Immunoreactive bands were scanned using UNSCANIT (Silk Scientific, Orem, UT).

### RNA analysis

Tissues from 12.5 dpc (n = 3 wt, 3 het, and 2 null) or 17.5 dpc (n = 3 wt, 2 het, and 2 null) chroioallantoic placenta were homogenized in Tri Reagent (Molecular Research Center, Inc., Cincinnati, OH). Seven μg of total RNA from each sample was loaded per well and separated on a 1.5% denaturing agarose gel. Separated RNA was transferred to nitrocellulose by capillary transfer and baked at 80°C for two hours. Membranes were prehybridized for 3 hours at 42°C in buffer (50% formamide, 5× SSC, 50 mM Na_2_PO_4_, 5× Denhardts, 0.1% SDS, 0.1 mg/ml salmon sperm DNA). Blots were then hybridized in the same buffer with radiolabeled probe.

A partial murine Isg15 or Ubp43 cDNA was synthesized using total RNA from pregnant murine uterine tissue and RT-PCR with the following primers; Isg15 anti-sense strand, 5'-ATGGCCTGGGACCTAAAGGTG; Isg15 sense strand, 5'-AAGCTCAGCCAGAACTGGTCT; Ubp43 anti-sense strand, 5'-ATGGGCAAGGGGTTTG-3'; and Ubp43 sense strand, 5'-TCAGGATCCAGTCTTC-3'. Amplicons were subcloned into ZeroBlunt (Invitrogen, Carlsbad, CA) vector and sequenced to confirm identity. Complementary DNA was radiolabeled using 50 μCi [α-^32^P]dCTP and Klenow in a standard random primer labeling reaction. Northern blots were washed 3×, 5 minutes each at 42°C (2× SSC/0.1% SDS or 1× SSC/0.1% SDS). Membranes were exposed to film for 6 days at -80°C. Northern blot results were normalized to an 18S rRNA band. Autoradiograms were scanned and quantitated using UNSCANIT.

### Bacterial and viral screening

To verify that the immune system of these mice was not compromised by bacteria or pathogens we submitted tissue samples to the following accredited facilities for analysis. Tissue samples from a breeding pair that was housed in our facility for six months were submitted to the Wyoming State Veterinary Laboratory (Laramie, WY) and serological samples were submitted to Charles River Laboratories (Wilmington, MA).

### Morphology of day 12.5 implantation sites

Murine implantation sites were fixed in 4 % paraformaldehyde, paraffin-embedded, serially sectioned at 6 μm and then stained with hematoxylin/eosin (H&E) for standard histological examination. Immunohistochemistry was performed with 20 μg/ml of Isolectin B4 antibody in 0.1% BSA-PBS (Vector Lab) and 20 μg/ml Horseradish Peroxidase Streptavidin (Vector Lab) as the enzyme conjugate. The staining was developed with AEC (Vector Lab) and counterstained with hematoxylin.

### Statistical analysis

To determine differences among genotypes and dpc, data were analyzed using the General Linearized Model of Statistical Analysis Systems (SAS, 1998). The effects of genotype on concentrations of and expression of free Isg15 and concentrations of conjugated Isg15 and VEGF were analyzed using one-way ANOVA, followed by protected preplanned *t-*test (P < 0.05) comparisons.

## Results

### Genotyping and genetic profile

Fetal development for Ubp43 -/-, -/+ and +/+ offspring is represented in Figure 1. Genotype ratio was 2:5:1 on day 12.5 and 2:2:1 on day 17.5. All null offspring were dead on day 17.5, whereas 75% of null offspring were dead on day 12.5. Several +/- offspring also died between 12.5 and 17.5 dpc. Chi square analysis revealed that genotypic ratios for live offspring on 12.5 (P < 0.05) and 17.5 dpc (P < 0.01) did not follow the normal expected mendelian ratio of 1:2:1.

### Expression of Isg15 and Ubp43 in fetal-derived placenta

**Figure 2:**
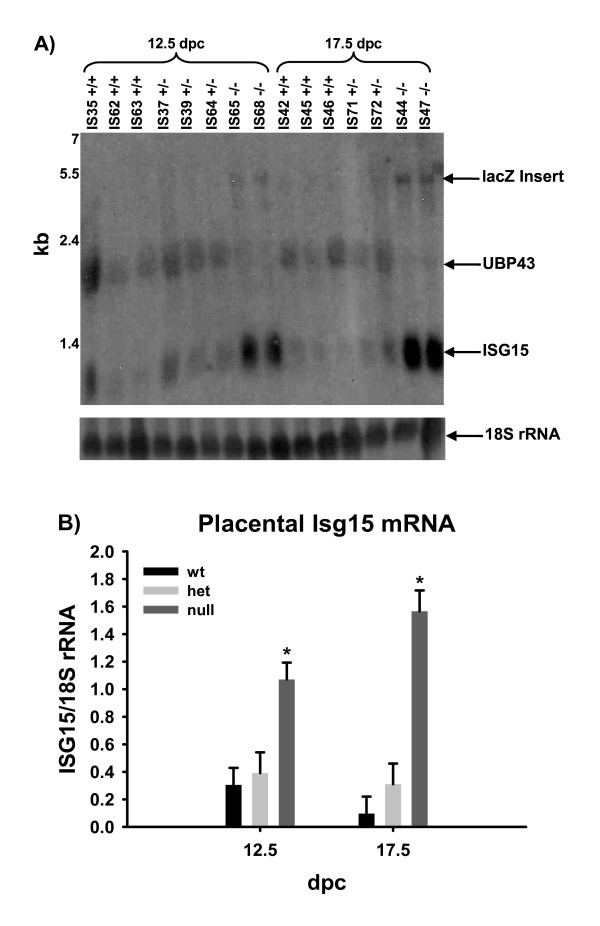
Northern blot analysis for Ubp43 and Isg15 (panel A). Fetal chorioallantoic placental RNA was analyzed for Ubp43 and Isg15 using cDNA probes. Isg15 mRNA was present on 12.5 and 17.5 dpc in all genotypes. Ubp43 mRNA was present in wt and het tissues at the correct size. 18S rRNA was not different. Panel B represents a graphical interpretation of the expression of Isg15 in fetal chorioallantoic placenta. Isg15 mRNA was greater in null derived placenta in comparison to wt or het placenta. Means are LSM ± SE and represent 3 +/+, 3 +/-, and 2 -/- on 12.5 dpc, and 3 +/+, 2 +/- and 2 -/- fetal chorioallantoic placenta. * denotes P < 0.07.

Northern blot analysis of CP tissue for Isg15 and Ubp43 verified expression of Isg15 in all genotypes (Figure 2). Ubp43 mRNA was only present in northern blots at the correct size within wt- and het-derived tissues. Null fetal-derived tissues containing the beta-galactosidase (lacZ) reporter had an expected shift in the size of the mRNA band of approximately 3 kilobases. Null CP tissue had greater (*P *< 0.07) expression of Isg15 mRNA regardless of dpc in contrast to wt or het (Figure 2). All samples were normalized to an 18S rRNA as a loading control.

### Protein expression of Isg15 in fetal and maternal tissues

**Figure 3:**
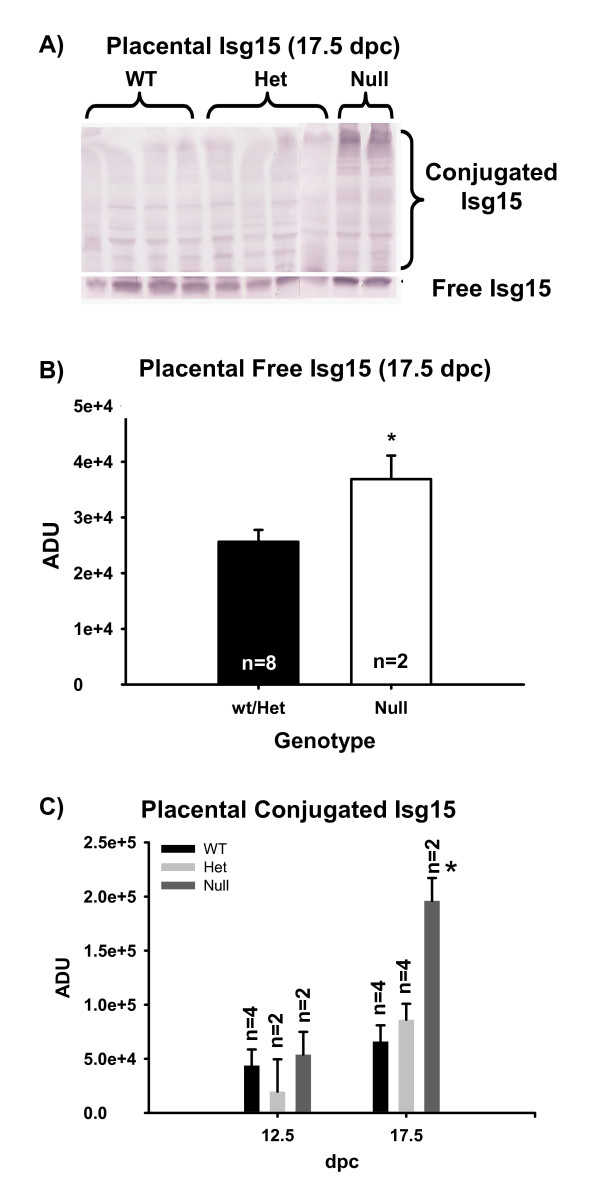
Fetal chorioallantoic placental concentrations of Isg15. Panel A represents a western blot of placental free Isg15 (~17-kDa) and conjugated Isg15 (> 30-kDa) on 17.5 dpc. Panel B represents quantitation of free placental Isg15. Panel C represents quantitation of conjugated placental Isg15. Null fetal-derived placental tissue had greater concentrations of Isg15 when compared to pooled wt/het placenta. An interaction (P < 0.05) of genotype by dpc suggested that null fetal-derived placental tissue had greater concentrations of Isg15 conjugates on 17.5 dpc in contrast to other genotypes or null tissue on 12.5 dpc. Means are LSM ± SE. * denotes P < 0.05.

Since Ubp43 regulates the conjugation state of Isg15 (free- or conjugated) we evaluated the influence of increased Ubp43 mRNA and subsequent Isg15 mRNA in Ubp43 -/- fetuses on the protein levels of Isg15 in fetal and maternal tissues. Fetal chorioallantoic placenta were analyzed by western blot for abundance of Isg15 and conjugated Isg15 proteins. Fetal placental concentrations of Isg15 were greater (*P *< 0.05) in null compared to pooled wt and het fetal-derived tissues on 17.5 dpc (Figure 3). Interestingly, an interaction occurred where concentrations of conjugated Isg15 proteins were greater (*P *< 0.05) in null CP on 17.5 dpc in contrast to all other genotypes on 12.5 or 17.5 dpc.

**Figure 4:**
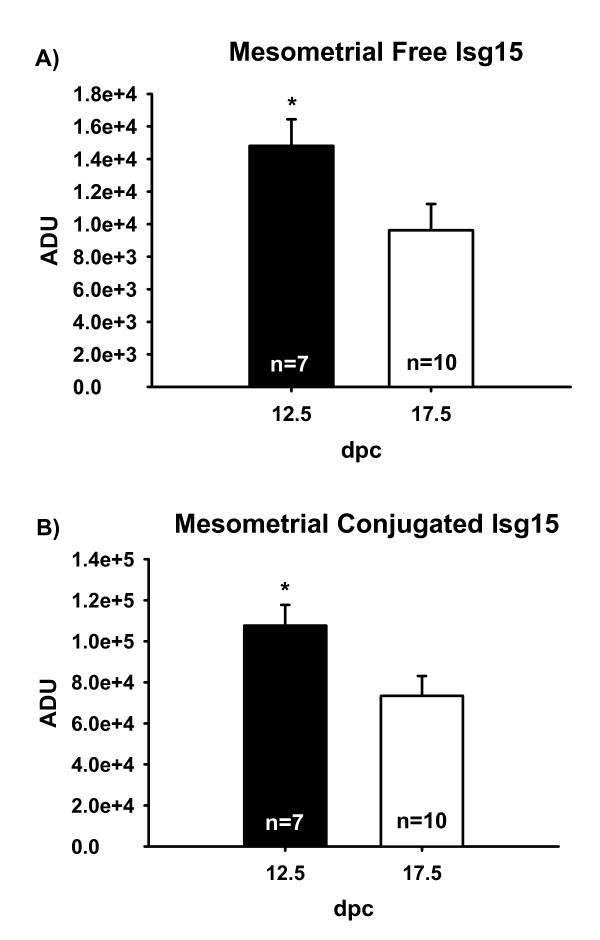
Mesometrial (MM) concentrations of free Isg15 (A) and conjugated Isg15 (B). Based on western blot detection of Isg15, MM had greater concentrations of Isg15 on 12.5 versus 17.5 dpc (A). MM also had greater concentrations of conjugated Isg15 on 12.5 dpc in contrast to 17.5 (B). Means are LSM ± SE. * denotes P < 0.05. Arbitrary densitometric units (ADU).

To determine if fetal Ubp43 deletion altered levels of free and conjugated Isg15 in maternal tissues, western blot analysis was also conducted using MM and AM derived tissue. Mesometrial tissue had greater (*P *< 0.05) Isg15 and conjugated Isg15 on 12.5 than on 17.5 dpc (Figure 4). Mesometrial concentrations of Isg15 or conjugated Isg15 did not differ among genotypes (data not shown).

**Figure 5:**
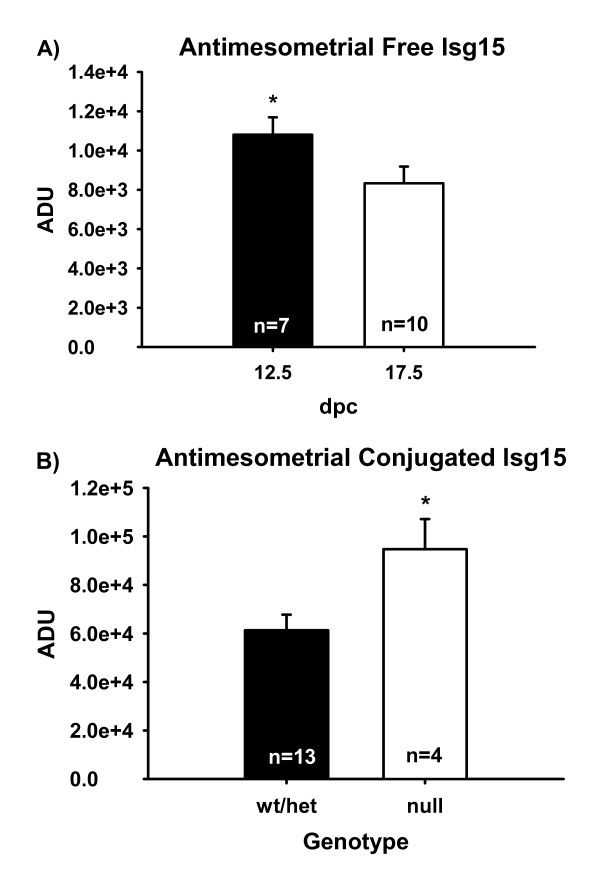
Anti-mesometrial (AM) tissue concentrations of free Isg15 (A) and conjugated Isg15 (B). Concentrations of Isg15 determined by western blot did not differ across genotype, but were greater on 12.5 dpc in AM tissue than on 17.5. AM concentrations of conjugated Isg15 did not differ across dpc. However, AM tissue had increased concentrations of conjugated Isg15 in tissue surrounding null fetuses in contrast to tissue overlying wt/het fetuses. Means are LSM ± SE. * denotes P < 0.05.

Anti-mesometrial tissue was also analyzed for free Isg15 and its conjugates by western blot. No interactions (dpc × genotoype) were observed in AM tissue for free or conjugated Isg15, therefore only main effects (dpc or genotype) are reported. Concentrations of AM Isg15 were greater in those tissues surrounding null fetuses regardless of dpc (data not shown). Overall concentrations of Isg15 in AM tissue were greater (*P *< 0.05) on 12.5 versus 17.5 dpc (Figure 5). No differences in conjugated Isg15 were seen between AM tissues surrounding wt or het fetuses, therefore data were pooled. Concentrations of conjugated Isg15 were greater (*P *< 0.05) in AM surrounding null fetuses in contrast to AM encompassing wt and het fetuses.

Comparison of MM versus AM tissue identified that free Isg15 was not different (P > 0.05) between the two tissues. However MM decidua in contact with fetal-derived placenta had greater (P < 0.05) levels of conjugated Isg15 in contrast to AM tissue (data not shown).

### Virological and bacterial analysis of mouse colony

To verify that fetal losses were not due to a compromised sanitary environment of the animal facility, maternal and fetal tissues from a Ubp43 heterozygous interbreeding were tested for a broad spectrum of bacteria and viruses (Wyoming State Veterinary Diagnostic Laboratory, Laramie, WY) and serum samples collected from a breeding pair were screened for viruses (Charles River Laboratories, Wilmington, MA). Both virological and bacteriological analyses were negative.

### Environmental influence of elevation on angiogenic and hypoxic markers in the placental compartment

**Figure 6:**
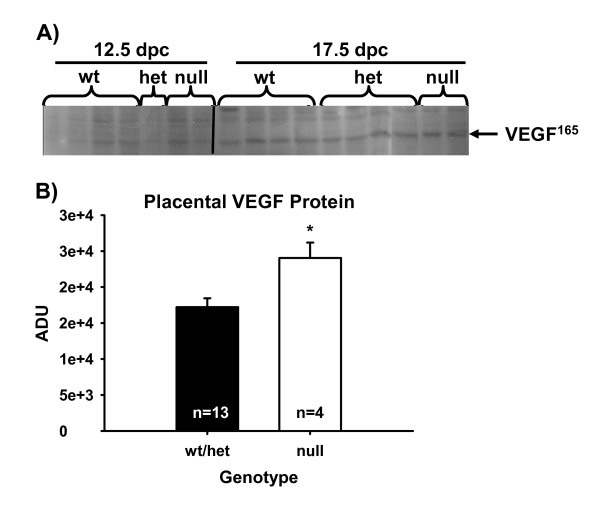
Concentrations of chorioallantoic placental VEGF^165 ^as determined by western blot (panel A). Concentrations of VEGF^165 ^were greater in null fetal placenta in comparison to pooled wt/het-derived placenta (panel B). Means are LSM ± SE. * denotes P < 0.05.

To test if fetal losses were due to the increased elevation of the facility at UW (approximately 2,183 m; when compared to sea level for the facility at The Scripps Research Institute), which may alter oxygen tension, fetal and maternal derived tissues were analyzed by western blot for various angiogenic markers such as VEGF, angiopoietin-1 and Flt-1 receptor or hypoxia markers such as GLUT-1 and AMP kinase-1alpha. No differences were seen within tissues based on dpc or genotype for the angiogenic markers – angiopoietin-1 or Flt-1 receptor or the hypoxic markers. However in CP tissue, concentrations of VEGF were greater in null fetal-derived tissue in comparison to pooled wt/het fetal-derived placental tissues (Figure 6A and 6B).

### Morphological evaluation of placental tissue

**Figure 7:**
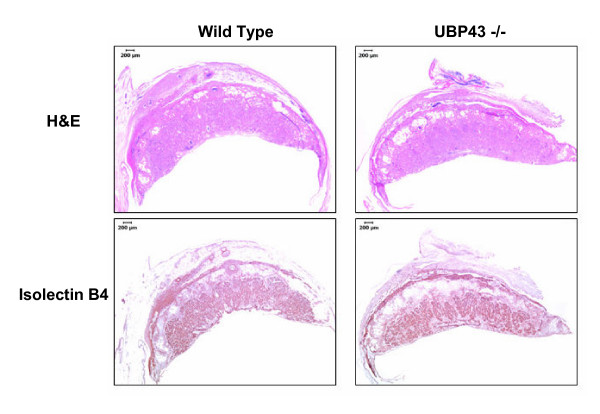
General morphology (hematoxolin-eosin; top panels) and labyrinthe endothelial cell staining (isolectin B4; bottom panels) in 12.5 dpc implantation cross sections. ____ = 200 μm.

Hematoxylin and eosin staining revealed that decidual and junctional or spongiotrophoblast zones appeared normal in 12.5 dpc implantation sites from Ubp43 wt fetuses. Gross evaluation of Ubp43 null mice consistently showed more densely packed cells within the labyrinth layer. Also the junctional layer was disrupted, which is not typical of a mid-gestation placenta. The decidual tissue of the null-derived placenta had less cellular compaction in contrast to the Ubp43 wt placenta. Ubp43 heterzygous fetuses had variable morphology in implantation sites that was not consistently similar to the wt or the null fetuses. Complementary characterization of the morphology of the junctional zone was performed using immunohistochemistry techniques for isolectin B4 (Figure 7, lower panels). Isolectin B4 has greater specificity for labyrinth-derived cells and leaves the junctional zone primarily devoid of stain [40]. Placental tissues derived from Ubp43 null fetuses had large infarcted areas in contrast to wt Ubp43 fetal-derived placentas.

## Discussion

Endometrial expression of Isg15 occurs during early pregnancy in several mammals; including the cow [3], ewe [4], sow [5] mouse [6,7,20]) and primates [1,20,41]. Interferon-stimulated gene product 15 is hypothesized to play an intrinsic role during implantation and placentation by forming an isopeptide bond with intracellular proteins, potentially altering their activity. An Isg15-specific protease, Ubp43, is also up-regulated by type I IFNs in a manner that is similar to up-regulation of Isg15 [42]. Therefore, it was postulated that regulation of Ubp43 within the placenta could play a significant role during pregnancy and fetal development.

Previous reports by our group [6] and others [7] described increased Isg15 mRNA within AM decidua when compared to deciduoma of psuedopregnant mice on 7.5 dpc. We also reported that AM decidual Isg15 mRNA increased from 4.5 to 7.5 and from 7.5 to 9.5 [6]. Expression of Isg15 mRNA was restricted to maternal tissue and was not evident in conceptus-derived tissue on 7.5 dpc. [43]. During later stages of pregnancy, MM decidua also expressed Isg15 and its conjugates on 12.5 and 17.5 dpc regardless of fetal Ubp43 genotype. The fact that free and conjugated Isg15 were present through 17.5 dpc in mice may suggest that not only is Isgylation necessary in the uterus during early pregnancy, it may also influence placental function, implantation and fetal development throughout pregnancy.

The level of Isg15 within the anti-mesometrial uterine tissue was greater on 12.5 dpc versus 17.5 dpc, which was similar to the temporal pattern of expression inMM decidua. However, Isg15 and its conjugates were increased in anti-mesometrial tissue surrounding the null fetuses in contrast to wild-type or heterozygous fetuses. Increased anti-mesometrial uterine Isg15 and conjugates surrounding null fetuses suggests that fetal deletion of Ubp43 can influence maternal endometrial levels of conjugated Isg15, even when tissues are not directly adjacent to one another.

Isg15 and its conjugates were also present in the vascular MM tissue surrounding the fetus and this level of expression was greater when compared to anti-mesometrial tissue on 12.5 and 17.5 dpc. As expected, there was an increase in conjugated Isg15 in null fetal-derived placental tissue in contrast to tissue from wild-type and heterozygous fetuses. This was probably caused by an accumulation of Isgylated proteins because of lack of action of the de-isgylating enzyme through Ubp43 gene deletion. Increased transcription of the Isg15 gene in response to Ubp43 -/- is more difficult to explain. However, this might be an indirect compensatory mechanism because of a lack of return of conjugated Isg15 to the free pool of Isg15. Because free Isg15 is essentially consumed, perhaps the cell responds to the Ubp43 null by increasing transcription of the Isg15 gene in order to continue to provide free Isg15 that can then enter the conjugating pathway.

Previous researchers have shown that deletion of Ubp43 in knockout mice that survived to term led to accumulation of Isg15 conjugates in the ependymal lining of the brain, whereas wild-type and heterozygous litter mates had undetectable levels of conjugated Isg15 proteins [30]. Null mice had an increased incidence of hydrocephalus and premature death suggesting that Ubp43 has an important role in postnatal brain development. Under our conditions we were never able to produce viable Ubp43 null offspring indicating that Ubp43 deletion may also play a major role in placental function and/or fetal development. Ubp43 deficient mice are hypersensitive to type I IFN and have enhanced and extended IFN signaling responses that lead towards augmentation of apoptotic responses [13]. The fetal Ubp43 -/- mice in our study may have been subjected to increased apoptotic activity as a result of the type I IFN hypersensitivity. Disregulation of Isgylation in Ubp43 deficient fetal mice may have caused fetal death due to enhanced activation of Stat 1 tyrosine phosphorylation, DNA binding and IFN-mediated gene activation [13]. Fetal death may also have been due to non-Isg15 responses such as a lack of direct interaction of Ubp43 with the IFN receptor [35].

Messenger RNA for Isg15 was increased in Ubp43 deleted placental tissue as well. Increased expression of Isg15 in the absence of Ubp43 suggests that Ubp43 may regulate Isg15 expression directly or have an indirect effect by influencing the level of free Isg15 within tissues. It is more likely that the latter is true since previous reports have indicated that lack of Ubp43 expression enhanced sensitivity to type I IFN signaling and led to increased expression of interferon-stimulated genes, including Isg15 [13,31,44].

Implantation sites appeared disrupted in Ubp43 null when compared to wt mice. The junctional zone and the decidua were less densely compact in Ubp43 null when compared to wt fetuses. This disregulation of Isg15 through deletion of Ubp43 is hypothesized to contribute to 75% fetal mortality on day 12.5 of pregnancy.

Vascular endothelial growth factor is upregulated in response to hypoxia during physiological conditions, including such events as wound healing [45,46]. Vascular endothelial growth factor is expressed and localized within trophoblast cells of various species [47-49] and is considered to be a very powerful mitogenic and angiogenic factor [50,51]. In vitro studies on VEGF^165 ^incubation with human trophoblast cells inhibited cell migration through an extracellular matrix chamber [52]. The increased levels of VEGF^165 ^in the placenta may inhibit appropriate implantation resulting in fetal mortality. Albeit, since the mortality rate was 75 and 100% on 12.5 and 17.5 dpc, respectively, the increased VEGF may be a result of fetal resorption activity or it may have increased in response to hypoxia. Der and co-workers [42] reported that VEGF-C mRNA is up-regulated in response to IFN-α and -γ. Therefore another possible cause for increased VEGF in null fetal-derived tissue may be an indirect result of increased sensitivity to IFNs due to Ubp43 deletion.

Furthermore Ubp43 -/- mice are hypersensitive to Type I IFN, implicating a role for Ubp43 to downregulate IFN responses (Ritchie et al., 2002). These investigators later identified that Ubp43 attenuated IFN signaling by direct interaction with the region of the IFNAR2 receptor subunit that interacts with JAK1 [35]. Subsequently interaction of Ubp43 to the IFNAR2 receptor suppressed JAK1 interactions and concomitantly decreased downstream phosphorylation cascades and other IFN-responsive signaling events. These actions of Ubp43 are independent of its effects on Isg15.

An expected Mendelian ratio of 1:2:1 wt:het:null live progeny from het interbreedings of Ubp43 knockout mice was achieved previously using C57 BL/6 × 129 background with the pGK-Neo fragment [30]. However, during colony expansion we were never able to produce any viable null offspring at the University of Wyoming (UW) facility by breeding het (C57 BL/6 × 129 crossed to Swiss Webster to remove the pGK Neo fragment) pairs. In addition to loss of null fetuses by 12.5 dpc, we also observed loss of +/- fetuses by 17.5 dpc. We estimate that the het animals at the UW facility had less than 50% Swiss Webster genetic contribution.

Ubp43 +/- interbreedings (C57 BL/6 × 129 × Swiss Webster; n = 10 litters) were followed to term, and from these offspring 16 were +/+ and 31 were +/-, implicating that the Mendelian ratio for offspring was 1:2 for wt:het, as would be expected when considering the lack of null offspring. Therefore, we recorded fetal genotype ratios on 12.5 and 17.5 dpc in utero to investigate the loss of null offspring. On 12.5 dpc we had a ratio of 2:5:1 from a total of 30 live and dead fetuses (4 litters). And on 17.5 dpc the ratio was 2:2:1 calculated from 35 live and dead fetuses (6 litters). On 12.5 dpc only 25% of the null fetuses were viable and by 17.5 dpc all null fetuses were non-viable. There also was a noticeable loss of +/- fetuses by 17.5 dpc. The loss of heterozygous fetuses by 17.5 dpc was unexpected and might be caused by a Ubp43 gene dosage due to loss of one allele. When considering live fetuses only, genotypes did not follow normal expected Mendelian ratios on 12.5 and 17.5 dpc.

The same mice at the Scripps Research Institute produced Ubp43 -/- mice from heterozygous breedings. At the age of genotyping (3–4 wks old), a ratio of 1:4:1 from 58 pups was observed. However, work done at Scripps found that backcrossing Ubp43 het mice (C57 BL/6 × 129) with C57 BL/6 mice to F10 generation did not produce any viable Ubp43 -/- pups. Similar to reports by the UW facility, homozygous Ubp43 null mice died in utero by 12.5 dpc. It is believed that genetic drift, influenced by the contribution from C57/BL 6 lineage, altered the outcome at the UW facility. The founder animals at the UW facility could have influenced the expressionicity of the +/- interbreedings by either increased genetic contribution from C57 BL/6 or potential deletion of the 129 pGK-Neo fragment by crossing into the Swiss Webster strain.

Differences in viable offspring due to location may be a reflection of facility environmental cues. However, bacterial and viral analyses of Ubp43 mice from our facility were negative suggesting that other causes may be responsible for fetal loss. The slight changes in angiogenic and hypoxia markers provided preliminary evidence that hypoxia played a major role in fetal loss, however future experiments are planned to further study this possibility. Mouse genetic background and associated genetic modifiers plays a significant role in sensitivity to interferon [53] and may also explain why fetuses died in the present experiment, but apparently were born and then died post-natally in other Ubp43 -/- experiments [30,38]. The genetic drift due to a mixed background may actually have caused the unexpected fetal loss.

## Conclusion

In summary, we have found that the Isg15 system was present in maternal and, for the first time reported, in fetal-placental tissues. As expected, wild-type and heterozygous fetal-derived tissue did not appear to differ or alter the maternal or fetal Isg15 system. Furthermore, Isg15 mRNA was present through late stages of pregnancy in fetal tissue. Ubp43 deletion was verified in null fetal tissue by genotyping and northern blot analysis. And, VEGF was the only angiogenic marker that was altered by Ubp43 deletion.

Deletion of the Ubp43 gene causes disregulation of the Isg15 system, a disrupted implantation site and fetal death in transgenic mice in the present study. This result is different from reports in the original Ubp43 -/- paper [30] and from a more recent UBP43/Isg15 double -/- paper [38] where no impact on reproduction was reported. However, work done at Scripps revealed that backcrossing Ubp43 het mice (C57 BL/6 × 129) with C57 BL/6 mice to the F10 generation did not produce any viable Ubp43 -/- pups and these fetuses also died by 12.5 dpc. For this reason, and because the Ubp43 -/- mice are from different genetic backgrounds, we suspect that there are strain specific genetic modifiers (not unlike human populations), that may impact the lethality of deletion of Ubp43.

Also, the original Isg15 -/- was not described to have any reproductive problems and also was not described to be involved with antiviral responses [36]. Since the original Isg15 -/- report, it has been demonstrated that deletion of Isg15 does indeed affect antiviral responses and survivability in response to influenza A/WSN/33 and influenza B/Lee/40 virus, herpes simplex virus type 1, gammaherpesvirus 68 and Sindbis virus infection [54]. Thus, there are differences in the Isg15 responses due to different viruses. And even though these investigators report no difference in reproductive phenotype, there may be strain differences as just described for the Ubp43 mice in addition to subtle environmental differences such as mild hypoxia due to locatioin of the mice. For example, we also have recently obtained the Isg15 -/- mice from the Knobeloch laboratory [38] and have observed a 50% fetal death rate by 12.5 dpc (our unpublished results), which provides support for the concept that this reproductive phenotype is influenced by genetic background and/or location.

It is concluded that proper regulation of the Ubp43 and Isg15 systems are required for fetal viability and development. Up-regulation of Isg15 conjugates in Ubp43 null fetuses may influence cellular responses and signal transduction within maternal and fetal components of the placenta, thereby altering proper implantation or function of the placental unit. Deletion of Ubp43 causes up-regulation of conjugated Isg15 in null fetuses, which may influence targeted proteins within maternal and fetal components of the placenta. Disruption of these target proteins may influence proper implantation or function of the placental unit causing deleterious effects on placentation and fetal development in Ubp43 null mice. Whether the deletion of Ubp43 induces an abnormal accumulation of conjugated Isg15 which then contributes to fetal lethality, or this is confounded or pre-empted by non-Isg15 actions such as Isg15-independent impact of Ubp43 on the IFN receptor [35] awaits further study.

## Authors' contributions

LR conducted the animal breeding, genotyping, and tissue harvesting. LR also carried out the molecular techniques including western blotting and northern analysis. LR prepared and statistically analyzed data, drafted the manuscript and assisted with editing. KA assisted with molecular techniques and editing. KR, MY, MS, and D-EZ created and provided the transgenic mice. D-EZ also provided guidance and insight during manuscript editing. LH conducted the immunohistochemistry. TH assisted with the design and statistical analyses of the experiments as well as writing and editing of the manuscript.
